# Senescence of lung mesenchymal stem cells of preterm infants by cyclic stretch and hyperoxia via p21

**DOI:** 10.1152/ajplung.00355.2023

**Published:** 2024-09-24

**Authors:** Judith Behnke, Maurizio J. Goetz, Lena Holzfurtner, Pauline Korte, Astrid Weiss, Tayyab Shahzad, Jochen Wilhelm, Ralph T. Schermuly, Stefano Rivetti, Saverio Bellusci, Harald Ehrhardt

**Affiliations:** ^1^Department of General Pediatrics and Neonatology, Justus-Liebig-University Giessen and Universities of Giessen and Marburg Lung Center (UGMLC), Member of the German Center for Lung Research (DZL), Giessen, Germany; ^2^Justus-Liebig-University Giessen and Universities of Giessen and Marburg Lung Center (UGMLC), Excellence Cluster Cardio-Pulmonary Institute (CPI), Member of the German Center for Lung Research (DZL), Giessen, Germany; ^3^Institute for Lung Health (ILH), Giessen, Germany; ^4^Division of Neonatology and Pediatric Intensive Care Medicine, Department of Pediatrics and Adolescent Medicine, University Medical Center Ulm, Ulm, Germany

**Keywords:** bronchopulmonary dysplasia, cellular senescence, hyperoxia, mechanical ventilation, p21

## Abstract

Phenotype distortion of lung resident mesenchymal stem cells (MSC) in preterm infants is a hallmark event in the pathogenesis of bronchopulmonary dysplasia (BPD). Here, we evaluated the impact of cyclic mechanical stretch (CMS) and hyperoxia (HOX). The negative action of HOX on proliferation and cell death was more pronounced at 80% than at 40%. Although the impact of CMS alone was modest, CMS plus HOX displayed the strongest effect sizes. Exposure to CMS and/or HOX induced the downregulation of PDGFRα, and cellular senescence preceded by p21 accumulation. p21 interference interfered with cellular senescence and resulted in aggravated cell death, arguing for a prosurvival mechanism. HOX 40% and limited exposure to HOX 80% prevailed in a reversible phenotype with reuptake of proliferation, while prolonged exposure to HOX 80% resulted in definite MSC growth arrest. Our mechanistic data explain how HOX and CMS induce the effects on MSC phenotype disruption. The results are congruent with the clinical observation that preterm infants requiring supplemental oxygen plus mechanical ventilation are at particular risk for BPD. Although inhibiting p21 is not a feasible approach, limiting the duration and magnitude of the exposures is promising.

**NEW & NOTEWORTHY** Rarefication of lung mesenchymal stem cells (MSC) due to exposure to cyclic mechanical stretch (CMS) during mechanical ventilation with oxygen-rich gas is a hallmark of bronchopulmonary dysplasia in preterm infants, but the pathomechanistic understanding is incomplete. Our studies identify a common signaling mechanism mediated by p21 accumulation, leading to cellular senescence and cell death, most pronounced during the combined exposure with in principle reversible phenotype change depending on strength and duration of exposures.

## Graphical abstract

**Figure:**
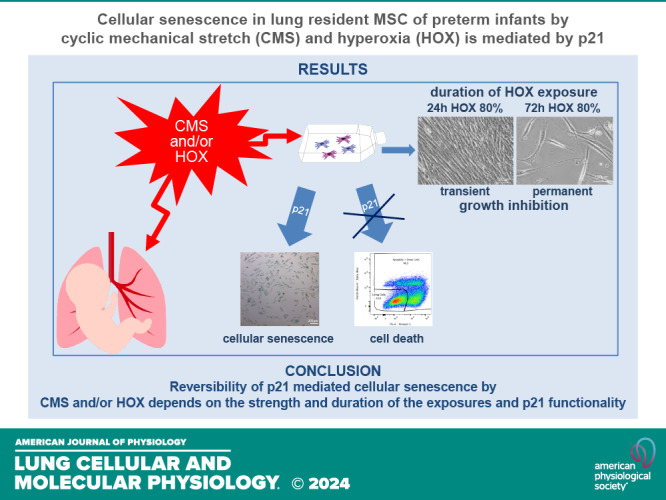

## INTRODUCTION

Bronchopulmonary dysplasia (BPD) remains one of the most devastating morbidities following premature delivery and imposes lifelong consequences on pulmonary function and quality of life (1). BPD has multifactorial origins and mechanical ventilation and oxygen toxicity constitute key disease drivers (2). Despite all the treatment advances in respiratory management during the last decades, the overall rate of BPD did not decline (3).

BPD pathology comprises alveolar airspace simplification, pulmonary vessel rarefication, and perturbed mesenchymal structures. Disturbed lung-resident mesenchymal stem cell (MSC) function constitutes a key event leading to BPD. Initial observations already at the turn of the last century associated the disruption of PDGFRα signaling with distortion of MSC functionality, impaired lung development, emphysema, and neonatal death (4–6). Lineage tracing of PDGFRα-positive cells gave primarily rise to alveolar myofibroblasts, and their abrogation led to alveolar simplification (7). Lastly, attenuation of PDGFRα signaling aggravated lung injury in newborn mice exposed to mechanical ventilation and hyperoxia (7, 8). Recent experimental evidence in rodents solidified the key role of MSC for alveolar epithelial but as well as for vascular development (7–11). However, in contrast to adult lung fibrosis models, experimental evidence revealed that, in the neonatal situation, PDGFRα-positive cells do not contribute to pathologic myofibroblasts (11, 12). It needs to be specified how mechanical ventilation with oxygen-rich gas induces the rarefication of PDGFRα positive cells in the neonatal lung, and which phenotype alterations occur in preterm lung-resident MSC, as can be observed during physiologic lung development for myofibroblast marker expression like α-SMA (7, 12, 13).

In vitro studies enabled further insights into this research issue. Lung MSC isolated from preterm rabbits exposed to oxygen or oxygen plus mechanical ventilation revealed less colony formation and differentiation capacity, and different gene expression of key lung regulatory processes, including cell cycle and cell division (14). Lung MSC isolated from newborn rats exposed to hyperoxia displayed altered proangiogenic effects and immunofunctionality (11). In line, the colony-forming potential, the phenotype, and the cellular functionality of MSC isolated from fetal lung tissues of 16 to 18 wk of gestation were altered following exposure to hyperoxia (15). These data are in line with previous studies on hyperoxia-induced growth inhibition in lung cell populations, and the induction of cellular senescence became evident even at low oxygen fractions of 40% (16, 17). In accordance with these results, previous observations in the newborn hyperoxia rodent models reported the detection of a marked p21 upregulation, which is one of the key proteins for the induction of cellular senescence, but a causal link to the process of alveolar simplification was not established (18). Prior studies on the functionality of p21 in newborn hyperoxia lung injury contrast the assumption of a disease-aggravating functionality by p21, as p21 knockout mice exposed to hyperoxia displayed increased acute lethality and an aggravated lung injury phenotype that became particularly evident when the follow-up period after hyperoxia exposure, between 2 and 6 wk of age, was studied, where alveolar enlargement became much more evident. This finding was attributed to a reduced ability to repair the lung injury (19).

Studies performed on MSC isolated from tracheal aspirates of ventilated preterm infants provided valuable insights into MSC pathology and confirmed the results on MSC dysfunctionality from rodent models (20, 21). MSC from infants with BPD displayed higher levels of phospho-GSK-3β/β-catenin, which correlated with the α-SMA content, representative for increased TGF-β signaling and a surrogate of premature myofibroblast differentiation (22). Autocrine TGFβ1 production was identified as one important mechanism of this phenotype alteration (23). Thereby, TGFβ1 counteracts pro-survival and pro-proliferative NF-κB function (21, 24). Furthermore, downregulation of PDGFRα was detected as another important characteristic of MSC pathology, and longitudinal studies in preterm infants undergoing prolonged mechanical ventilation with oxygen-rich gas documented the further suppression of PDGFRα receptor levels (8, 25).

Here, we extended our previous studies on lung-resident MSC from preterm infants (21) and mimicked the clinical situation of mechanical ventilation with oxygen-rich gas by applying cyclic mechanical stretch (CMS) and exposure to hyperoxia (HOX). Besides the consequences for cell viability, particular focus was set toward changes in functional properties within a unique in vitro setting. The clinical situation of infants with less and more severe perturbation of gas exchange was simulated by exposure to 40% and 80% of oxygen, which segregated distinct lung phenotypes in the preclinical in vivo models (26–28).

## MATERIALS AND METHODS

### Cell Culture and Experimental Approach to Lung Resident MSC from Preterm Infants

MSC cultures were established from tracheal aspirates of preterm infants <30 wk of gestation (demographics given in Table 1 and Supplemental Tables S1 and S2) as described previously (8, 21). Cells were kept in constant growth and experiments were started between passage 3 and 6. MSC were seeded in DMEM (41965092, Thermo Fisher, Waltham, MA) supplemented with 10% fetal calf serum (FCS) (10270106, Thermo Fisher, Waltham, MA), 2% penicillin-streptomycin (15140122, 10,000 IU/mL–10,000 µg/mL, Thermo Fisher, Waltham, MA), and 2% gentamycin (03928174, 40 mg/mL, Ratiopharm, Ulm, Germany) on collagen type I-coated flexible-bottom culture plates (BioFlex, Flexcell International Corporation, Burlington, NC). Parallel settings of unstretched controls in normoxia and exposures to CMS and/or HOX for 72 h were started the next day when cells had reached a confluency of 10–25% (21). CMS was executed using the FX-5000 Tension System (Flexcell International Corporation, Burlington, NC) in sine shape with elongation with a minimum of 1% and a maximum of 8% at a frequency of 1 Hz with duty cycle of 40%. The maximum applied static stretch reflected tidal volumes of 10 mL/kg body weight. We were not able to respect oscillatory stretch as a correlate of spontaneous breaths. HOX was applied at 40% or 80% of oxygen, relying on self-constructed chambers and constant oxygen concentrations with a maximum variation of ±5% that were monitored using a GOX 100 oxygen sensor (Greisinger electronic GmbH, Regenstauf, Germany). siRNA transfection experiments were performed as described before (21) using Lipofectamine RNAiMAX (13778150, Thermo Fisher, Waltham, MA) and siRNA against p21 (4390824-s417, Silencer Select siRNA, Thermo Fisher, Waltham, MA) or negative control (4390843, Silencer Select Negative Control siRNA, Thermo Fisher, Waltham, MA) at a concentration of 8.3 nM (25 pmol/6well) 24 h before the start of experimental procedures. Cellular senescence staining was performed using the kit from Cell Signaling (9860S, Senescence β-Galactosidase Staining Kit, Cell Signaling, Danvers, MA) according to the manufacturer’s instructions. All experiments with human MSC were performed in accordance with the principles of the Helsinki Declaration and approved by the ethics committee of the Justus-Liebig-University Giessen (Az. 135/12). Written informed parental consent was obtained from the parents of all participating infants. Trial registration at Deutsches Register Klinische Studien (DRKS00004600).

**Table 1.** Demographics of the study collective (n = 17)

Variable	Mean (IQR) or *n* (%)
GA, wk	24 + 3 (23 + 1–25 + 5)
BW, g	652 (460–720)
Male	9 (52.9)
Multiples	9 (52.9)
SGA	5 (29.4)
Chorioamnionitis	3 (17.6)
ANCS	11 (64.7)
IMV, days	28.35 (18–36.5)
Cultivation, day of life	16 (11.0–18.5)
IVH (any grade)	1 (5.9)
ROP (any stage)	13 (76.5)
FIP	4 (23.5)
NEC	0 (0.0)
BPD (any severity) mild moderate severe	16 (94.1) 4 (23.5) 3 (17.6) 9 (52.9)
Death (before discharge)	1 (5.9)

Perinatal and outcome characteristics of the *n* = 17 preterm infants whose resident lung MSC cultures were used in the study. Data are presented as mean (IQR; interquartile range) or *n* (%). ANCS, antenatal corticosteroids; BPD, bronchopulmonary dysplasia; BW, birthweight; FIP, focal intestinal perforation; GA, gestational age; IMV, invasive mechanical ventilation; IVH, intraventricular hemorrhage; NEC, necrotizing enterocolitis; ROP, retinopathy of prematurity; SGA, small for gestational age.

### Cell Expansion Index

The cell expansion index (CEI) was calculated as the quotient of (living cell count at the end of the experiment/living cell count at the start of the experiment) using manual cell counting via Hemocytometer (Neubauer chamber) for each intervention group. Dead cells were identified by trypan blue staining. The change in CEI due to the exposures of CMS and/or HOX was calculated compared with the spontaneous proliferation as (CEI exposure/CEI control) – 1. Thus, in our experimental setting, the CEI reflects the increase in the number of living cells from the start to the end of the observation (Supplemental Fig. S3).

### Flow Cytometry

Flow cytometry was conducted on a BD FACSAria III Cell Sorter (Becton Dickinson, Franklin Lakes, NJ); Diva software (version 6.1.3, Becton Dickinson, Franklin Lakes, NJ) was used for data acquisitions and data was analyzed with FlowJo (software version 10.7.1, Becton Dickinson, Franklin Lakes, NJ). Cell counting beads (ACFP-100-3, Spherotech, Lake Forest, IL) were used for MSC quantification. We performed cellular phenotyping using the following fluorochrome-labeled anti-human antibodies for surface staining, according to the manufacturer’s instructions: CD146 (361008, BioLegend, San Diego, CA, conjugation: PE-Cy7), CD90 (328114, Biolegend, San Diego, CA, conjugation: APC), CD73 (562430, Becton Dickinson, Franklin Lakes, NJ, conjugation: BV421), CD45 (560777, Becton Dickinson, Franklin Lakes, NJ, conjugation: V500), and CD165 (392040, Ancell, Bayport, MN, conjugation: FITC). Isotype-matched control antibodies were ordered from the same companies. The staining time for surface antibodies or isotypes was 30 min, followed by washing with staining buffer (1 × PBS/1% Albumin V; 70011036 Thermo Fisher, Waltham, MA; 80764, Carl Roth, Karlsruhe, Germany). Staining was performed on ice and MSC incubated with primary antibodies at the recommended concentrations. Apoptosis induction was determined relying on an Annexin V (640947, PE conjugated, BioLegend, San Diego, CA)/SYTOX Blue (S34857, Thermo Fisher, Waltham, MA) life/dead double staining and using a life/dead fixable kit (423109, Zombie Red Fixable Viability Kit, BioLegend, San Diego, CA). MSC were washed and resuspended in PBS and in Annexin V Binding Buffer (422201, BioLegend, San Diego, CA). Cells were incubated with count beads and Annexin V for 30 min, and immediately before counting SYTOX blue dye was added to exclude dead cells. For quantification absolute apoptosis was calculated as the quotient of (apoptotic and dead cells/frequency of single cells).

### Microarray and Signaling Pathway Analyses

RNA was isolated with the RNeasy Mini Kit (74104, Qiagen GmbH, Hilden, Germany), and MSC were treated according to the manufacturer’s instructions. Purified total RNA was amplified and Cy3-labeled using the LIRAK kit (Agilent Technologies, Santa Clara, CA) following the kit instructions. Per reaction, 200 ng of total RNA was used. The Cy3-labeled aRNA was hybridized overnight to 8 × 60K 60-mer oligonucleotide spotted microarray slides (Agilent Technologies, design ID 072363). Hybridization and subsequent washing and drying of the slides were performed following the Agilent hybridization protocol. The dried slides were scanned at 2 µm/pixel resolution using the InnoScan is900 (Innopsys, Carbonne, France). Image analysis was performed with Mapix (software version 8.2.7), and calculated values for all spots were saved as GenePix results files. Stored microarray data were evaluated using the R software (R Foundation for Statistical Computing, Vienna, Austria) and the LIMMA package (29) from Bioconductor (30). Mean spot signals were background corrected with an offset of 1 using the NormExp procedure on the negative control spots. The logarithms of the background-corrected values were quantile-normalized (29–31). The normalized values were then averaged for replicate spots per array. From different probes addressing the same National Center for Biotechnology Information (NCBI) gene ID, the probe showing the maximum average signal intensity over the samples was used in subsequent analyses. Genes were ranked for differential expression using a moderated *t* statistic (29). Pathway analyses were performed using gene set tests on the ranks of the *t* values (29). KFO 903 Core unit “Genome signatures and integrated systems biology of pathogen-host interaction and cellular networks in the infected and injured lung.”

The microarray data discussed in this publication have been deposited in NCBI’s Gene Expression Omnibus (GEO) (32) and are accessible through GEO Series accession number GSE235562 (https://www.ncbi.nlm.nih.gov/geo/query/acc.cgi?acc=GSE235562).

### Western Blotting

Cells were lysed with RIPA lysis buffer (24948, Santa Cruz Biotechnology, Dallas, TX) according to the manufacturer’s instructions. Protein concentration was quantified by Pierce BCA Protein Assay Kit (23225, Thermo Fisher, Waltham, MA) and measured via Nanodrop. After denaturation with Laemmli buffer containing 2-mercaptoethanol for 5 min at 95°C, the protein extract was loaded on a 10% SDS-gel. Electrophoresis was performed for 1 h at 40 mA. Protein transfer onto a nitrocellulose membrane (0.2 µm pore size) was done using a Transblot Turbo system (Bio-Rad, Hercules, CA) for 30 min (25 V, 1 A). After blockade of unspecific binding with 5% dry milk in PBS + 2.5% Tween (TBS-T) at room temperature for 1 h, the primary antibody was incubated overnight at 4°C in the blocking buffer. After three washes with TBS-T, the membrane was incubated with the respective HRP-labeled secondary antibody at room temperature for 1 h followed by another TBS-T buffer washing cycle. Membranes were subjected to immunoblot analyses using primary antibodies against αSMA (58669, Santa Cruz, Dallas, TX, mouse-monoclonal, 1:5,000), PDGFRα (3174S, Cell Signaling, Danvers, MA, rabbit monoclonal, 1:1,000), p21 (2947S, Cell Signaling, Danvers, MA, rabbit monoclonal, 1:1,000) and GAPDH as housekeeping protein (MAB37437, Merck, Darmstadt, DE, mouse monoclonal, 1:5,000). HRP-conjugated anti-rabbit (7074S, Cell Signaling, Danvers, MA, 1:1,000) and anti-mouse (sc-516102, Santa Cruz, Dallas, TX, 1:1,000) were used as secondary antibodies. Chemiluminescence was activated with SuperSignal West Femto kit (A38554, Thermo Fisher, Waltham, MA). Gels were detected with the ChemiDoc XRS+ Gel Imaging System (Bio-Rad, Hercules, CA). Comparison between different gels relied on an internal standard deposited on each gel and signal quantification was performed using ImageLab (version 6.0, Bio-Rad, Hercules, CA). The lane normalization factor was calculated as the quotient of (observed signal of housekeeping protein for each lane/highest observed signal of housekeeping protein on the blot), and the adjusted total band volume was normalized to GAPDH as the quotient of (observed experimental signal/lane normalization factor). All gels were normalized to control-intervention group. The order of samples in some blots was rearranged for the clearness of presentation without any further manipulation (indicated by separated boxes). Detection of the protein of target and of GAPDH control was executed on the same membranes. Original Western blot data are provided in the supplemental material (Supplemental Fig. S5).

### Statistical Analysis of in Vitro Exposures

For the screening of the effects of CMS and HOX on MSC phenotype, a sample size of 6 was calculated using a two-sided paired *t* test with a two-sided type one error of 0.05 and α power of 0.85. To account for dropouts due to insufficient experimental quality, the sample size for screening was increased by 33% to 8 primary MSC cultures.

For all experimental settings besides the microarray analyses, data are presented as mean and SE Student’s *t* test and one-way RM ANOVA were used to test for statistically significant differences. Statistical analyses were performed using SPSS (version 27.0, IBM SPSS Statistics, Armonk, NY) and Sigma Plot (version 12.3, Systat Software, San Jose, CA). Scatterplots were designed in GraphPad Prism (version 10.0, GraphPad Software, Boston, MA). Differences were considered significant at *P* values <0.05.

## RESULTS

We refined our in vitro model to study changes in lung-resident MSC phenotype and functionality in response to the combined application of CMS and HOX (8, 21). We exposed early passage MSC cultures from preterm infants with a gestational age (GA) between 22 and 30 wk (mean 24 + 3 wk GA, IQR 23 + 1–25 + 5 wk GA) to CMS and/or HOX for 72 h (MSC patient characteristics are detailed in Table 1 and Supplemental Tables S1 and S2). CMS was set as applied to the lung during conventional mechanical ventilation, and HOX was set at concentrations of 40% or 80% to reflect the clinical situation of a moderately or severely compromised gas exchange (8, 28).

### Cyclic Mechanical Stretch and Hyperoxia Lead to Reduced Viability and a Phenotype Change in MSC

**Figure 1.**
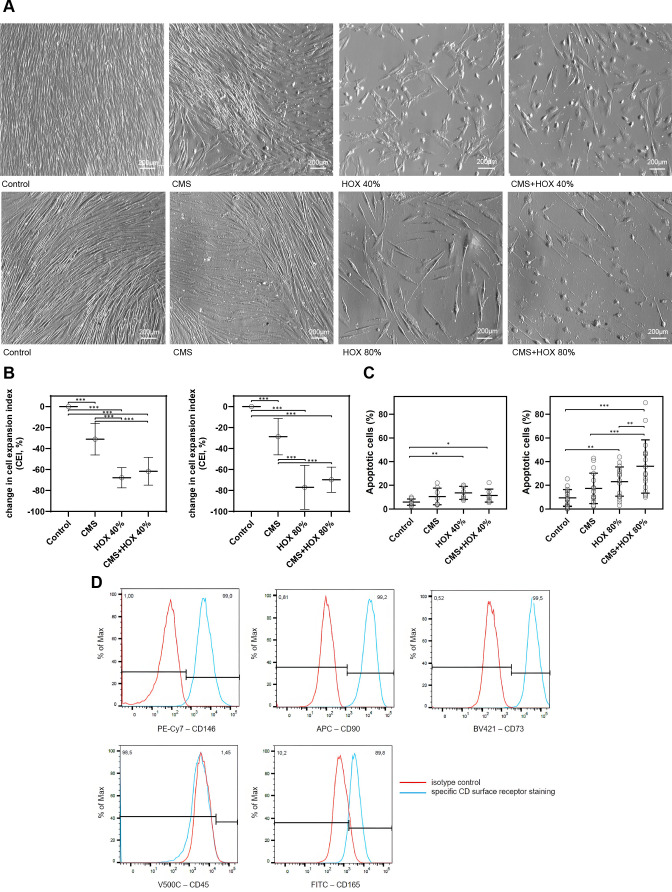
Single and combined treatment effect of cyclic mechanical stretch (CMS) and hyperoxia (HOX) on lung resident mesenchymal stem cell (MSC) proliferation of preterm infants. CMS and HOX lead to reduced viability and a phenotype change in MSC. *A*: bright-field microscopy of proliferation after 72 h of selective and combined exposure to CMS and/or hyperoxia (HOX 40% *top* or HOX 80% *bottom*). *B*: change in cell expansion index (CEI). CMS and/or hyperoxic exposure (HOX 40% *left* and HOX 80% *right*) led to a dose-dependent inhibition of MSC proliferation. *n* = 7 different MSC cultures. *C*: percentage increase of absolute apoptosis induced by selective and combined treatment with CMS and/or HOX (HOX 40% *left* and HOX 80% *right*). HOX 40% *n* = 8 MSC cultures, HOX 80% *n* = 17 MSC cultures. *D*: characterization of CD surface markers of resident MSC isolated from tracheal aspirates of preterm infants. Multicolor flow cytometry revealed expression of MSC-characteristic surface markers CD146, CD90 and CD73 whereas the hematopoietic marker CD45 was absent and CD165 weakly positive. Positive fraction of stained cells vs. isotype. Red = unstained control, blue = specific staining. APC, allophycocyanin; BV421, Brilliant Violet 421; FITC, fluorescein isothiocyanate; PE-Cy7, phycoerythrin-cyanine 7.

When MSCs were exposed to CMS or HOX 40% or 80% for 3 days, the spontaneous proliferation was notably reduced, which was already detectable in representative microscopic images and confirmed by manual cell counting. This confirmed the impact on cell proliferation from the start to the end of the exposure, but not the proliferative capacity at the end of the experiment (Fig. 1, *A* and *B*, and Supplemental Fig. S3). In addition, cell viability decreased after the application of CMS and/or HOX 40% or 80% (Fig. 1*C*). When comparing both HOX concentrations, the effect size was more pronounced for HOX 80%. The impact of CMS was lower than that of HOX both on proliferation and cell viability, and the largest effect size was seen when CMS plus HOX 80% were simultaneously applied.

**Figure 2.**
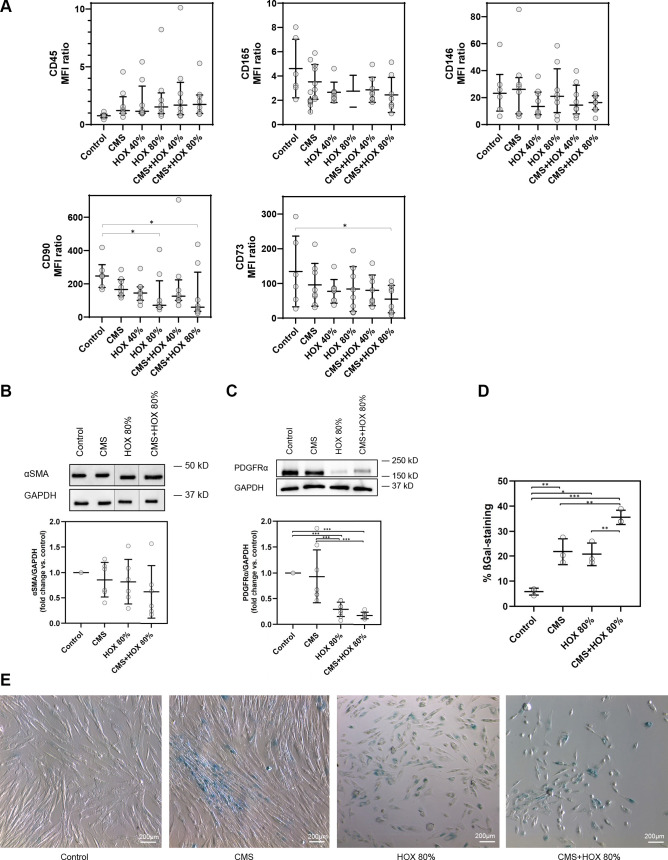
Surface and intracellular alterations in lung resident mesenchymal stem cells (MSC) induced by cyclic mechanical stretch (CMS) and hyperoxia (HOX). *A*: hyperoxic exposure (HOX 80%) and/or CMS plus HOX 80% reduced the expression of CD73 and CD90 surface marker expression. MFI ratio log10 (specific stain/control). *B* and *C*: intracellular phenotype alterations in lung resident MSC of preterm infants after exposure to cyclic mechanical stretch (CMS) and/or hyperoxia (HOX). *B*: Western blot analysis showed no significant change in α-SMA expression after selective and combined treatment with CMS and HOX 80% for 72 h. *n* = 6 different MSC cultures. The order of samples was rearranged for the clearness of presentation without any further manipulation (indicated by separated boxes). The bar graphs show the densitometry results expressed as the ratio of adjusted total band volume normalized to GAPDH and control. *C*: level of PDGFRα was reduced by CMS and HOX 80% whereby HOX 80% and HOX 80% plus CMS demonstrated the largest effect size after 72 h treatment. *n* = 8 different MSC cultures. The bar graphs show the densitometry results expressed as the ratio of adjusted total band volume normalized to GAPDH and control. *D* and *E*: CMS and/or HOX 80% increased cellular senescence-associated β-galactosidase (SA β-Gal) activity (blue; bright-field microscopy) after 72 h of exposure. Representative experiment of *n* = 3. Bar graph shows mean percentage of β-Gal positive cells of all experiments. Data are expressed as means (or median) ± SD (or IQR), *P < 0.05, **P < 0.01, ***P < 0.001, by one-way RM ANOVA and Bonferroni-correction.

Next, we aimed at characterizing changes in MSC phenotype provoked by CMS and/or HOX. Since the effect sizes were more pronounced for HOX 80%, we decided to focus on the more toxic scenario representing the clinical exposure scenario of infants at particular risk for severe BPD. Characterization of CD surface markers by multicolor flow cytometry revealed the expression of MSC-characteristic surface markers CD146, CD90, and CD73, whereas the hematopoietic marker CD45 was absent, and CD165 weakly positive (Fig. 1*D*). Flow cytometric analysis revealed a significant change in CD surface marker expression for CD73 and CD90 by the exposure to HOX 80% and/or CMS + HOX 80% (Fig. 2*A* and Supplemental Table S3) (15). When looking at classical further marker proteins of lung MSC, we did not detect a significant change in α-SMA expression after CMS and/or HOX (Fig. 2*B*) as we had observed after exposure to inflammatory stimuli previously although the α-SMA expression was reduced in five out of six samples (21). However, the level of PDGFRα was reduced by HOX and CMS plus HOX at a similar effect size (Fig. 2*C*). The highly attenuated proliferation of MSC by HOX together with previous publications on this topic, suggested studying cellular senescence in this context (16–18). Both CMS and HOX induced cellular senescence, which was again most pronounced for CMS plus HOX (Fig. 2, *D* and *E*).

### Cyclic Mechanical Stretch and Hyperoxia Activate p21 Signaling in MSC

**Figure 3.**
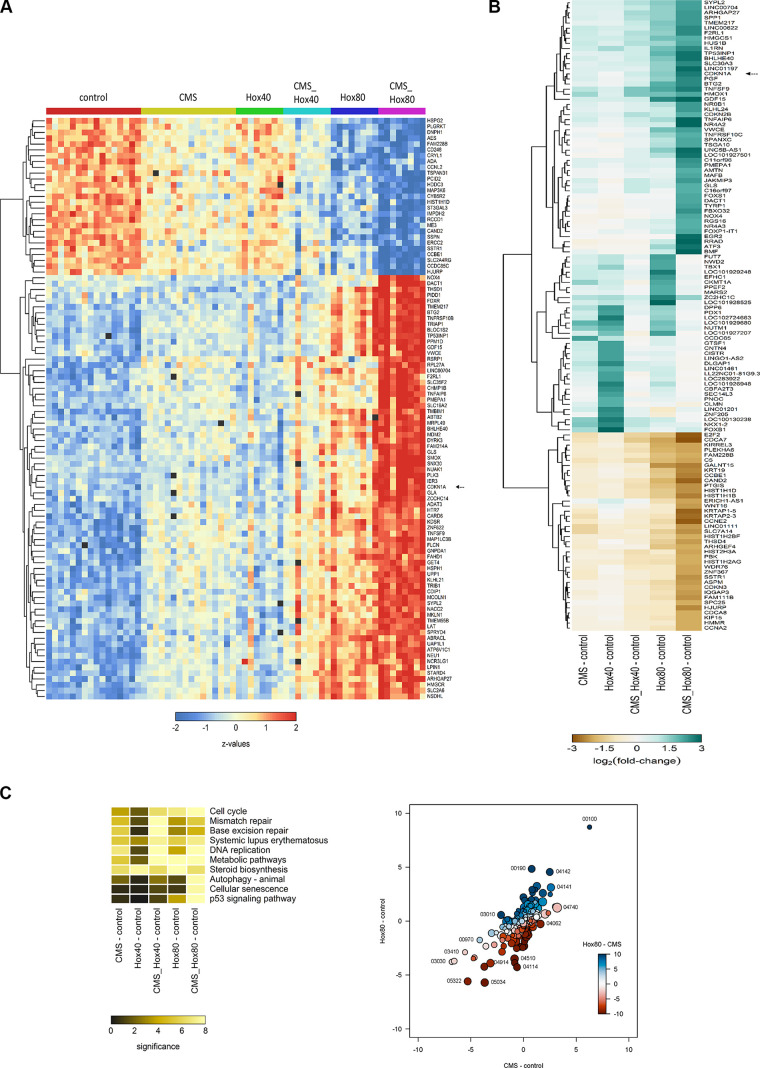
Cyclic mechanical stretch (CMS) and hyperoxia (HOX) activate p21 signaling in lung resident mesenchymal stem cell (MSC) of preterm infants. *A*: heatmap of the expression profiles for the 100 genes with highest F-statistics following 24 h of exposure to CMS and/or HOX 80%. Colors encode row-wise *z*-scores. Black boxes indicate missing values (spots excluded from the analysis because of structural artifacts). Genes with most prominent differential expression and smallest signal-to-noise ratio are selected, *n* = 16 samples. *B*: heatmap of the average log2 fold-changes against control of all genes being regulated at least twofold and in the same direction in all comparisons against the control. *C*, *left*: heatmap of the statistical significance of pathway perturbation by treatment of the 10 pathways with the highest statistical significance. The significance values are −log10(*P* values) from gene set tests performed on the ranks of the gene-wise moderated *t* statistics. Higher values (brighter colors) indicate lower *P* values, that is, a statistically more significant perturbation of the expression of the genes in the pathway based on enrichment score. *Right*: bubble plot summarizing results from KEGG pathway analyses. All axes show the *signed* statistical significance of the perturbation from the gene set tests. The sign indicates the sign of the direction of the perturbation. For instance, positive values on the y-axis indicate a stochastically higher expression of the genes under HOX 80% than under control conditions, negative values indicate a lower expression under HOX 80% than under control conditions. The colors indicate the signed significance from the gene set tests comparing the expression under HOX 80% than and control conditions directly. Each bubble represents one KEGG pathway, the size of the bubble is proportional to the number of genes in the pathway. Some outer bubbles were annotated with the respective KEGG pathway ID.

**Figure 4.**
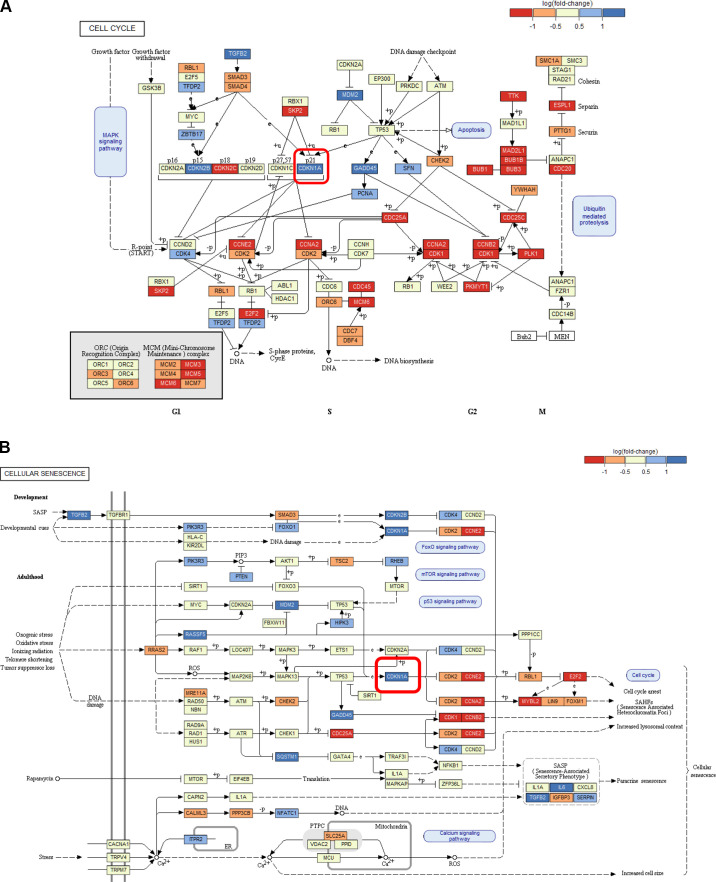
Pathway analysis of lung resident mesenchymal stem cells (MSC) exposed to cyclic mechanical stretch (CMS) and hyperoxia (HOX). *A*: colored map of the KEGG pathway 04110 (cell cycle). Control vs. CMS_Hox80. Boxes indicate genes, colors represent their differential expression. Pathway map provided by Kaneshi laboratories. The highlighted box indicates CDKN1A (p21). *B*: colored map of the KEGG pathway 04218 (cellular senescence). Control vs. CMS_Hox80. Boxes indicate genes, colors represent their differential expression. Pathway map provided by Kaneshi laboratories. The highlighted box indicates CDKN1A (p21).

**Figure 5.**
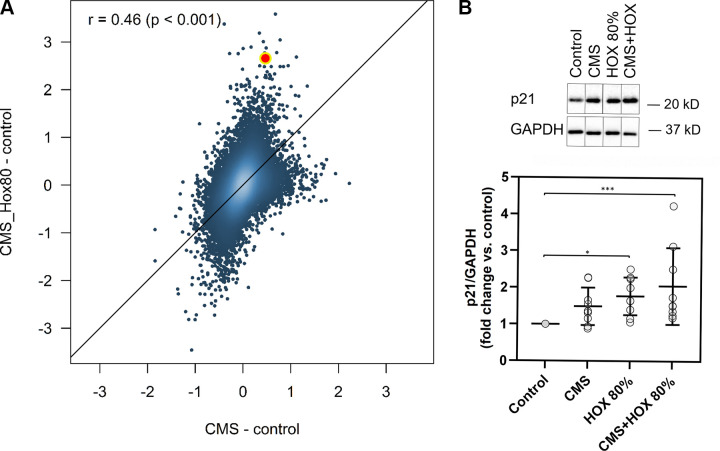
Regulation and activation of p21 in lung resident mesenchymal stem cells (MSC) by cyclic mechanical stretch (CMS) and hyperoxia (HOX). *A*: correlation of log2 fold-changes vs. control of CMS and CMS_Hox80 treated cells. Each dot represents an individual gene. The color gradient indicates the spatial density of the dots. The diagonal line is the identity line. Most genes are regulated in the same direction under both treatments (Pearson's *r* = 0.46, *P* < 0.001), with a generally stronger regulation by CMS_Hox80. The highlighted point indicates CDKN1A (p21). *B*: representative western blot analysis of p21 activation after 24 h exposure to CMS and HOX 80%. CMS and HOX induced the accumulation of p21 whereas more pronounced for HOX and CMS+HOX, *n* = 9 MSC cultures. The order of samples was rearranged for the clearness of presentation without any further manipulation (indicated by separated boxes). The bar graphs show the densitometry results expressed as the ratio of adjusted total band volume normalized to GAPDH and control. Data are expressed as means ± SD, *P < 0.05, ***P < 0.001, by paired *t* test.

To decipher the underlying signaling pathways and mechanisms of action, MSC subjected to CMS and/or HOX were analyzed by microarray analyses and prevailed a tremendous impact of both CMS and HOX on gene regulation (Fig. 3, *A* and *B*, and Supplemental Figs. S1 and S2). The role of genes regulating the cell cycle stood out (Fig. 3*C*). Figure 3*C* summarizes the results from the comparative analysis of the Kyoto Encyclopedia of Genes and Genomes (KEGG) pathway analyses by gene set tests. The pattern maps to a high positive overall correlation of the differential expression profiles, with a slightly stronger perturbation under HOX than under CMS. The pathways showing the strongest positive changes under both conditions compared with control, 00100, 00190, 04142, and 04141, are associated with energy and protein metabolism and ER stress, indicating a general stress response. Pathways showing the strongest negative changes, e.g., 05322, 05034, 04914, 03030, are associated with chromatin organization and DNA integrity. CDKN1A/p21 was identified as the most abundantly regulated gene (Fig. 4, *A* and *B*, Fig. 5*A* and Supplemental Figs. S1 and S2). These results were confirmed by Western blot analysis. Congruent with the functional and phenotype data, the effect of CMS alone was modest, and the accumulation of p21 following HOX and CMS plus HOX exposure reached statistical significance (Fig. 5*B*).

These data argue for p21 as a driver of cellular senescence induction by CMS and/or HOX. To confirm the molecular mechanism, inhibition studies were executed in the next step with particular focus on cell viability.

### Inhibition of p21 Augments Apoptosis Induction by Cyclic Mechanical Stretch and Hyperoxia in MSC

**Figure 6.**
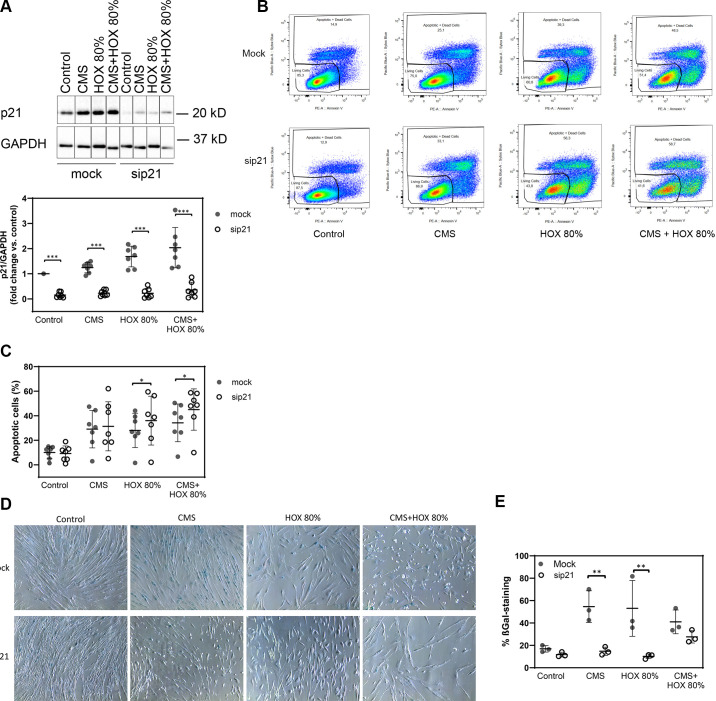
Inhibition of p21 augments apoptosis induction by cyclic mechanical stretch (CMS) and hyperoxia (HOX) in lung resident mesenchymal stem cell (MSC) of preterm infants. *A*: representative Western blot analysis of sip21 transfected cells shows inhibited accumulation. *n* = 7 samples. The order of samples was rearranged for the clearness of presentation without any further manipulation (indicated by separated boxes). The bar graphs show the densitometry results expressed as the ratio of adjusted total band volume normalized to GAPDH and control. *B*: representative flow cytometry analysis of each intervention group shows increase of apoptotic cell fraction in sip21 transfected MSC for each treatment with CMS and/or HOX 80%. Live/dead staining via Annexin V/SYTOX Blue flow cytometry analysis, absolute cell death (%) = dead cells/frequency of single cells. *C***:** inhibition of p21 augments apoptosis induction by CMS and HOX. When MSC with silenced p21 were exposed to CMS and/or HOX 80%, apoptosis induction was significantly increased and the increase in cell death was most pronounced for the combined intervention group. *n* = 7 samples. *D* and *E*: fraction of cells with induction of cellular senescence was reduced in MSC with silenced p21. One representative out of three experiments (blue; bright-field microscopy) Bar graph shows mean percentage of β-Gal positive cells of all experiments. Data are expressed as means ± SD, *P < 0.05, **P < 0.01, ***P < 0.001 by paired *t* test.

p21 accumulation following CMS and/or HOX 80% was efficiently inhibited by RNA interference in MSC (Fig. 6*A*). When MSC with silenced p21 were exposed to CMS and/or HOX 80%, cell death induction was significantly increased and was most pronounced for CMS plus HOX 80% (Fig. 6, *B* and *C*). During silencing p21, the fraction of cells with induction of cellular senescence by CMS or HOX 80% was significantly reduced but not completely abolished due to residual p21 (Fig. 6, *D* and *E*, and Supplemental Fig. S4). The higher fraction of mock-transfected cells in cellular senescence compared to Fig. 2*E* can probably be attributed to the transfection process and variability between individual MSC cultures.

These mechanistic insights specify the signal transduction via p21 as a critical step of CMS and HOX action in lung resident MSC of preterm infants.

### Reversibility of Growth Inhibition Induced by Cyclic Mechanical Stretch and Hyperoxia in MSC Depending on Exposure Strength and Duration

**Figure 7.**
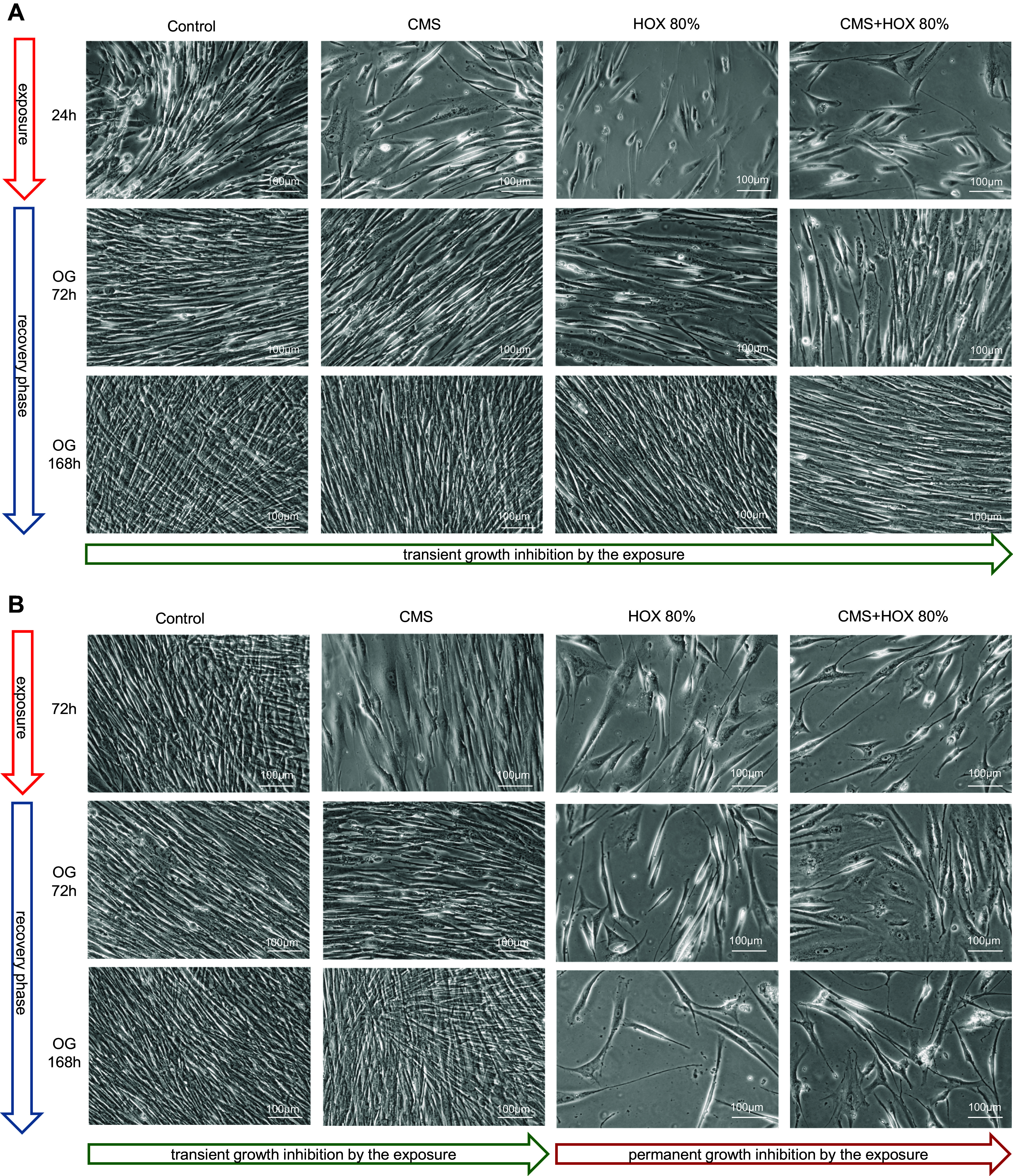
Reversibility of growth inhibition induced by cyclic mechanical stretch (CMS) and hyperoxia (HOX) in lung resident mesenchymal stem cells (MSC) depending on strength and duration of the exposure (HOX 80%). *A*: bright-field microscopy of proliferation after 24 h of selective and/or combined exposure to CMS and HOX 80%. Outgrowth (OG) of cells in all intervention groups after further 3 and 7 days of recovery in room air without CMS. *B*: bright-field microscopy of proliferation after 72 h of selective and/or combined exposure to CMS and HOX 80%. OG of cells only in the CMS selective treatment group but not in any of the HOX groups after 3 and 7 days of recovery in room air without CMS. Representative experiments of *n* = 3.

**Figure 8.**
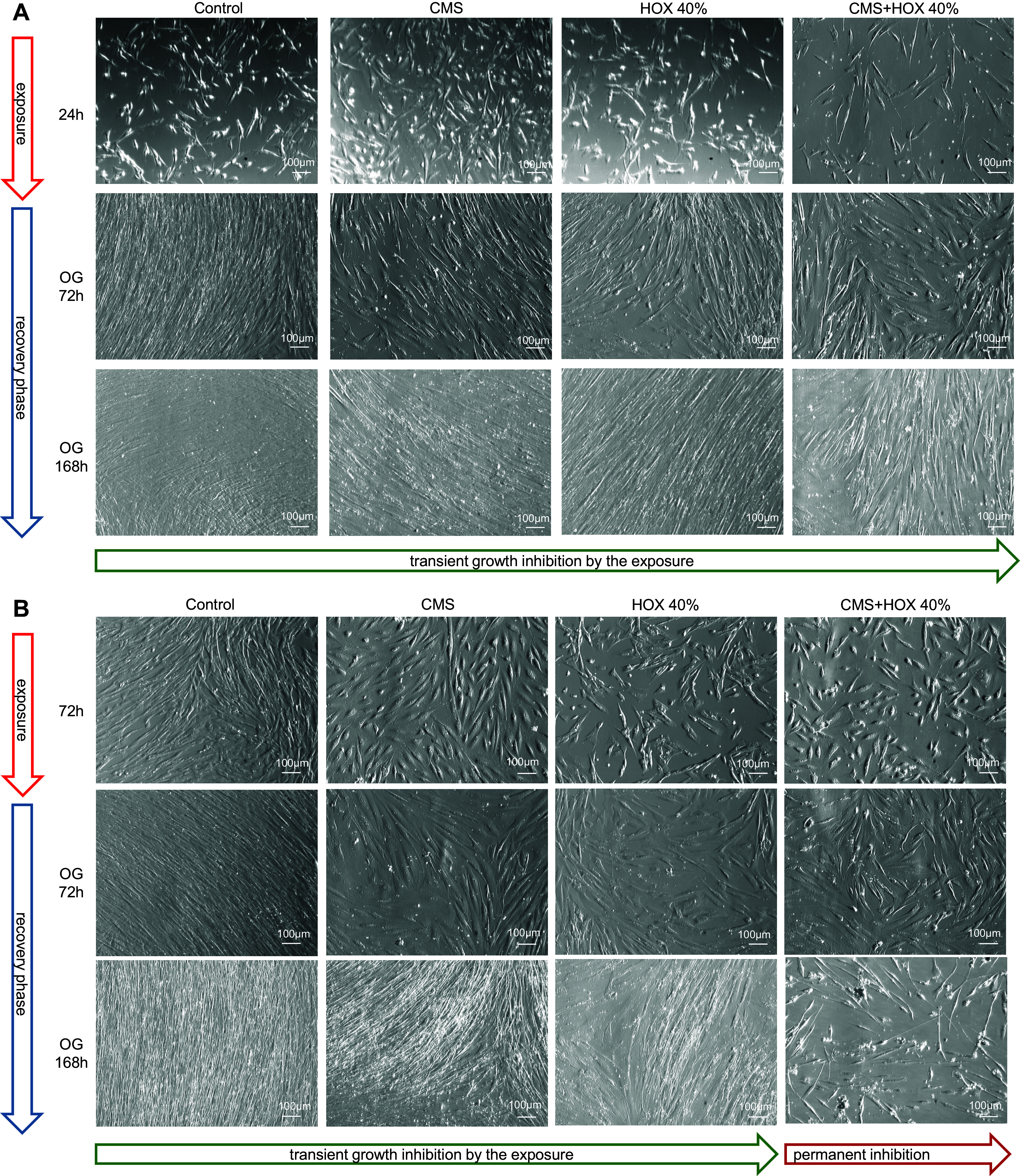
Reversibility of growth inhibition induced by cyclic mechanical stretch (CMS) and hyperoxia (HOX) in lung resident mesenchymal stem cells (MSC) depending on strength and duration of the exposure (HOX 40%). *A* and *B* show corresponding results as in Fig. 7, *A* and *B* respectively with HOX 40% with slightly weakened effect and preserved outgrowth (OG) after 72 h exposure to HOX but not CMS plus HOX. Representative experiments of *n* = 3.

**Figure 9.**
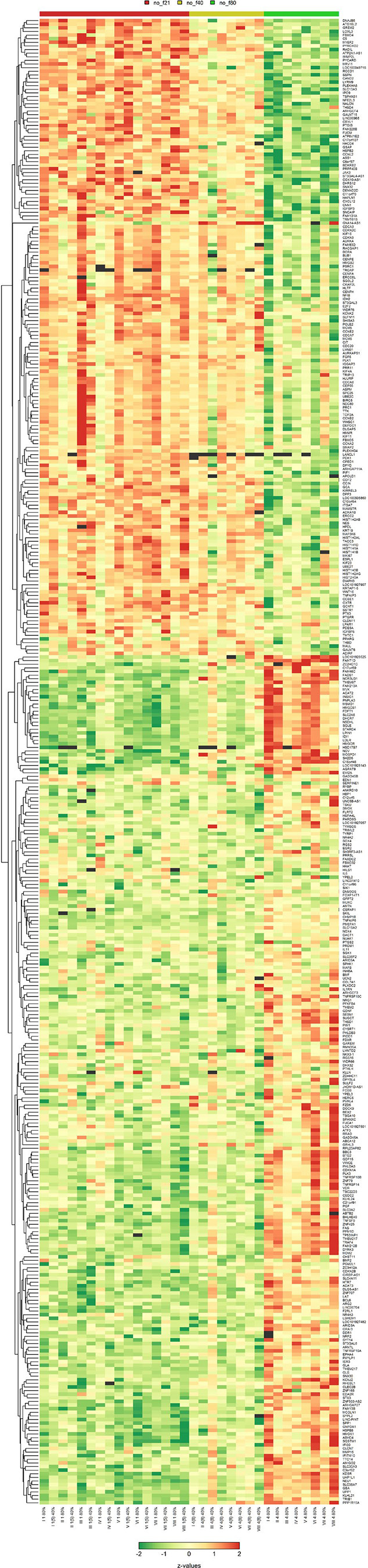
Expression profiles of genes in lung resident mesenchymal stem cells (MSC) after exposure to hyperoxia (HOX). Heatmap of the expression profiles for the 100 genes with highest *F* statistics following 24 h of exposure to HOX 40% or HOX 80%. Colors encode row-wise *z*-scores. Black boxes indicate missing values (spots excluded from the analysis because of structural artifacts). Genes with most prominent differential expression and smallest signal-to-noise ratio are selected, *n* = 32 samples.

**Figure 10.**
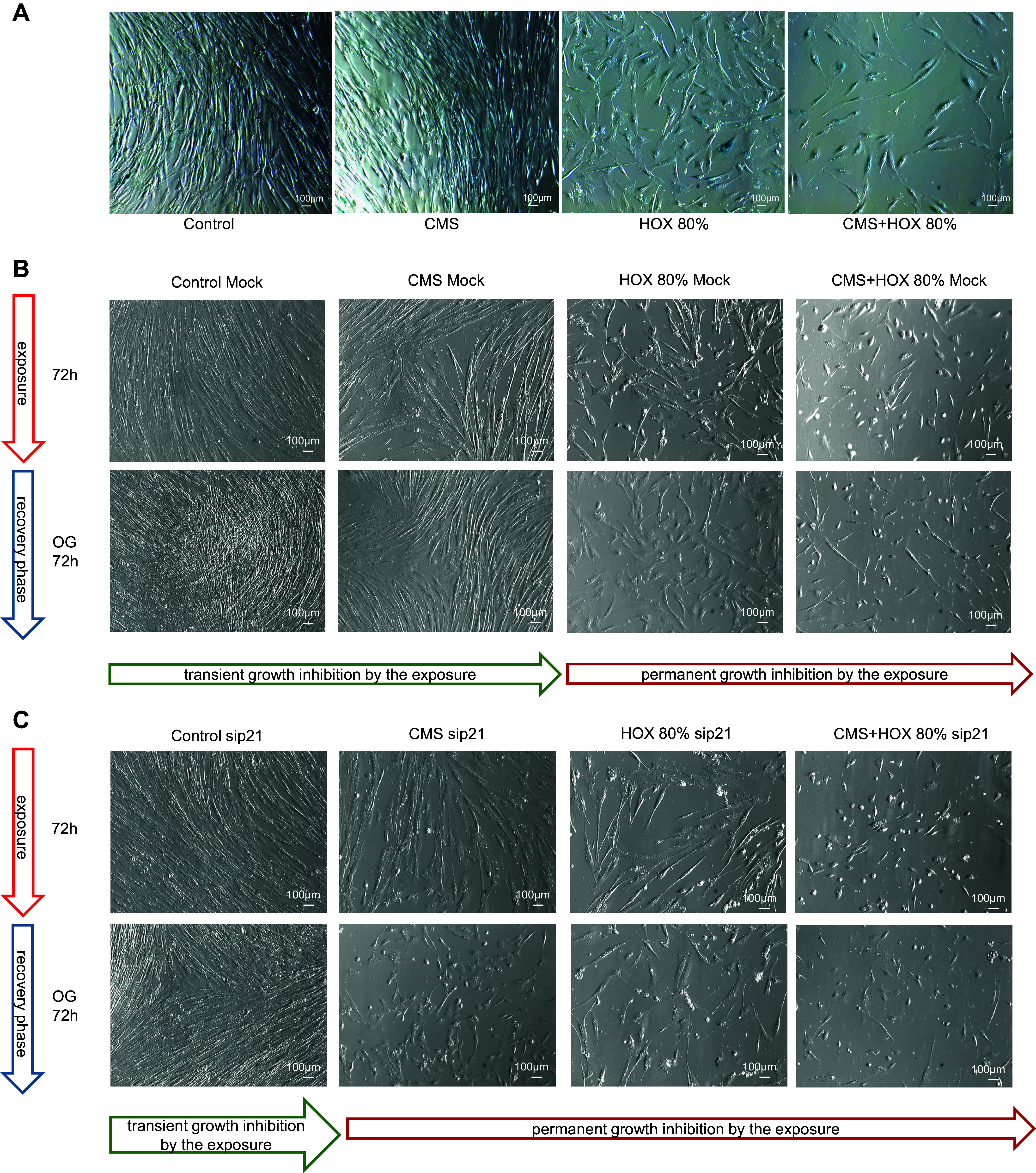
Cellular senescence and outgrowth of lung resident mesenchymal stem cells (MSC) exposed to cyclic mechanical stretch (CMS) and hyperoxia (HOX) after recovery in room air. *A*: after the recovery period, cellular senescence induction of MSC was reduced in the CMS group, while exposure to HOX 80% was still stained with permanent growth inhibition. Representative experiments of *n* = 3. *B* and *C*: bright-field microscopy of proliferation after 72 h of selective and/or combined exposure to CMS and HOX 80% after mock (*B*) or p21 (*C*) transfection. Outgrowth (OG) of cells occurred in MSC with silenced p21 only in the control group but not in any of the intervention groups after 3 days of recovery in room air without CMS. Representative experiments of *n* = 3.

To decipher the reversibility of the phenotype alterations exposed to CMS and/or HOX, we next exposed MSC to CMS and/or HOX 80% for 24 or 72 h followed by a recovery period in room air without CMS for up to further 7 days. Although MSC displayed only modest features of growth arrest after 24 h, the growth inhibition after exposure to CMS and/or HOX 80% for 72 h was more easily detectable by light microscopy (Fig. 7, *A* and *B*). Subsequent recovery enabled the further outgrowth of MSC in both situations for CMS while only the exposure to HOX 80% and CMS plus HOX 80% for 24 h enabled renewed outgrowth but not when cells were treated for 72 h. For 24 h exposure experiments complete well coverage was reached faster during the recovery phase than for the exposures for 72 h. Corresponding results were generated for HOX 40% with slightly weakened effects and preserved outgrowth after 72 h exposure to HOX 40% but not to CMS plus HOX 40% (Fig. 8, *A* and *B*). In line with the permanent growth arrest after HOX 80%, heatmap analyses prevailed a much higher impact of HOX 80% on gene regulation than seen for HOX 40% (Fig. 9). After the recovery period, cellular senescence in MSC was reduced in the CMS group indicating the principle reversibility, while it persisted unchanged after the exposure to HOX 80% and CMS + HOX 80% (Fig. 10*A*). Lastly, we studied the impact of p21 silencing on the renewed outgrowth of MSC. In contrast to the mock-transfected cells, RNA interference against p21 resulted in permanent growth inhibition in MSC exposed to CMS alone (Fig. 10, *B* and *C*).

These data allow the conclusion that the CMS and HOX induced phenotype alterations are in principle reversible even after periods up to 72 h, depending on the strength and duration of the exposures and the functionality of p21 that determine the reversibility.

## DISCUSSION

Actual treatment recommendations for the care of the preterm infant require postnatal exposure to a hyperoxic environment compared with the intrauterine situation to safeguard survival. Depending on the severity of gas exchange perturbation, the application of lower or higher fractions of oxygen together with mechanical ventilation, may be required to reach the therapeutic goals. Therefore, oxygen exposure and mechanical ventilation constitute inevitable exposures to the immature lung of preterm infants. Their detrimental action for BPD pathology has been dissected in detail during the last decades (2, 33). Disruption of lung MSC functionality has been identified as a hallmark event in this process. By establishing a novel in vitro model for the combined application of CMS and HOX, we provide causal evidence of how CMS and HOX induce phenotype alterations in lung resident MSC isolated from tracheal aspirates of preterm infants. Not surprisingly, the effects were more pronounced when HOX was applied at high concentrations and most devastating when HOX was combined with CMS and prolonged duration of the exposures. However, our data on the further outgrowth of MSC after discontinuation of CMS and HOX argue for a therapeutic opportunity when the strength and duration of CMS and HOX exposure can be limited. Corresponding therapeutic approaches are currently being investigated with high priority in clinical settings, with the aim to avoid intubation and mechanical ventilation and to reduce the oxygen toxicity (2, 33–35).

Our data provide the molecular link of how CMS and HOX disrupt MSC viability, proliferation, and phenotype. At first sight, our data might be surprising, but looking beyond neonatal lung injury the induction of cellular senescence via p21 has been established as a prevalent mechanism of HOX injury (36–38). Our results complement the previous publications on cellular senescence by HOX in newborn rodent models wherein cellular senescence was detected in lung fibroblasts and airway smooth muscle cells, and p21 accumulation was identified as a highly pronounced alteration following HOX exposure to the newborn rodent lung (16–18). In line, MSC from human fetal lungs displayed growth alterations when exposed to hyperoxia (15). However, our data on the increased apoptosis induction in MSC following inhibition of p21 argue for a cellular defense mechanism that is in principle reversible. This interpretation of our data is founded by the studies in p21 knockout mice where lung regeneration after hyperoxia was markedly reduced. Unfortunately, the authors were not prepared to study cell death induction during HOX or the number and distribution of MSC in the lungs after regeneration at that time (19). Here, we provide evidence that p21 accumulation protects the resident lung MSC from cell death induction by CMS and/or HOX and cellular senescence seems to be the responsible mechanism. Unfortunately, we were not able to specify the precise effects of silencing p21 on cell cycle distribution due to the increase in cell death induction following the exposures. Furthermore, we were not able to execute clonogenic assays on these primary cells what prohibits the definite determination of permanent growth inhibition.

The phenotypic characterization of the MSC after CMS and HOX revealed downregulation of PDGFRα as the second prominent alteration besides the induction of cellular senescence. This finding fits as well into the published literature where rarefication of PDGFRα expression and functionality in the immature lung following HOX was described as a key event (4–6, 8). Previous investigations of MSC obtained from ventilated preterm infants for PDGFRα expression revealed a reduced level in infants with BPD compared to the non-BPD group and decreased migration upon stimulation with PDGF isoforms (25). The PDGFRα level gets further suppressed after prolonged mechanical ventilation with oxygen rich gas (8). Our data further confirm the link between HOX and PDGFRα function in MSC and extend the action on PDGFRα expression to CMS. The data presented here reveal a MSC phenotype distinct from that detailed in our recent investigation on MSC phenotype alterations in the context of BPD (21). There, we did exclusively study infants with the diagnosis BPD and the variation in PDGFRα expression was probably too weak to deliver a clear distinction as was now induced by the strong exposure to 80% of oxygen. We previously observed a distinct MSC phenotype with increased proliferation and reduced expression of α-SMA upon stimulation with pro-inflammatory cytokines like IL-1β and TNF-α in vitro. Here, CMS and HOX changed the phenotype into the opposite direction with inhibition of MSC proliferation, cellular senescence and cell death induction and the number of samples studied might have been too small to detect a significant effect on α-SMA. It will be of utmost importance to bring these different stimuli together and to evaluate the combinatorial action as many of these infants are exposed to oxygen toxicity and inflammatory stimuli. Here, our recent data comparing HOX injury in vitro in precision cut lung slices (PCLS) and in vivo in newborn mice might shed some light on this issue. The developmental arrest and the reduction in PDGFRα positive cells in the lung were less pronounced when the immune system was removed before HOX exposure as it was done in the PCLS setting (27). Thus, we would argue for an additional adverse effect by the inflammatory response. When looking at the results from the two-hit models of prenatal amniotic infection and postnatal HOX, the augmentation of lung injury by inflammation together with HOX provide further evidence concerning this matter (39, 40).

As the MSC obtained from preterm infants had been exposed to CMS and HOX before initiation of our studies, they might have experienced a persistent change in phenotype as we described before for the cultivation of primary MSC for several passages in cell culture and upon one-time stimulation with pro-inflammatory cytokines (21). This might be the explanation for why we did not see a change in CD91 expression after HOX in contrast to the results obtained in untouched fetal MSC. Though these cells derived from the pseudoglandular stage of lung development while our cells originated from the late canalicular and early saccular stage between 22 and 30 wk of gestation and alterations in characteristics during lung development are frequent events (15). It must be stated that the effects of CMS and HOX exposure observed here rather confirm the results of our previous study on the longitudinal course in MSC phenotype of preterm infants. There, downregulation of PDGFRα was detected with prolonged mechanical ventilation with supplemental oxygen (8).

Taken together, our data add novel mechanistic insights into the knowledge on CMS and HOX-induced lung injury in the preterm infant. They provide further evidence of how PDGFRα downregulation and dysfunctionality are induced in lung MSC, which constitutes a hallmark event in the pathogenesis of BPD. Furthermore, our data indicate that the disruption of the intracellular signaling cascade leading to the phenotype distortion does not constitute a therapeutic option; rather, it suggests that research efforts need to be focused on restoring PDGFRα functionality or should be expanded to a broader scope of action to substitute the secretion of the panel lung growth factors that get depressed upon CMS and HOX (2, 8). The clinical relevance of our findings lies in the fact that they confirm a time frame of opportunity when the strength and duration of toxic exposures gets limited, and thereby the functionality of resident MSC as key drivers of lung growth get preserved (41–43). The implications of our study go far beyond the neonatal period and the data should encourage lung researchers faced with hyperoxic lung injury or the need for lung regeneration across the total lifespan to take our findings into consideration.

## DATA AVAILABILITY

Microarray data have been deposited in the National Center for Biotechnology Information (NCBI) Gene Expression Omnibus (GEO) database under accession number GSE235562 (https://www.ncbi.nlm.nih.gov/geo/query/acc.cgi?acc=GSE235562).

## SUPPLEMENTAL MATERIAL

10.6084/m9.figshare.26321464.v1 Supplemental Figs. S1–S5 and Supplemental Tables S1–S3: https://doi.org/10.6084/m9.figshare.26321464.v1.

## GRANTS

This work was supported by von Behring-Röntgen Foundation (65-0019, J.B.) and Clinical Research Unit KFO 309/2 (H.E.).

## DISCLAIMERS

The funder had no role in the design and conduct of the study, collection, management, analysis and interpretation of the data, preparation, review, or approval of the manuscript; and decision to submit the manuscript for publication.

## DISCLOSURES

No conflicts of interest, financial or otherwise, are declared by the authors.

## AUTHOR CONTRIBUTIONS

J.B. and H.E. conceived and designed research; J.B. and J.W. performed experiments; J.B., J.W., and H.E. analyzed data; J.B.J.W., and H.E. interpreted results of experiments; J.B., J.W., and H.E. prepared figures; J.B. drafted manuscript; J.B., J.B., M.J.G., L.H., P.K., A.W., T.S., J.W., R.T.S., and S.R. edited and revised manuscript; J.B., M.J.G., L.H., P.K., A.W., T.S., J.W., R.T.S., and S.R. approved final version of manuscript.
